# Principal Component Analysis Method with Space and Time Windows for Damage Detection

**DOI:** 10.3390/s19112521

**Published:** 2019-06-02

**Authors:** Ge Zhang, Liqun Tang, Licheng Zhou, Zejia Liu, Yiping Liu, Zhenyu Jiang

**Affiliations:** School of Civil Engineering and Transportation, State Key Laboratory of Subtropical Building Science, South China University of Technology, Guangzhou 510640, China; zhangge13756010981@163.com (G.Z.); lqtang@scut.edu.cn (L.T.); tcypliu@scut.edu.cn (Y.L.); zhenyujiang@scut.edu.cn (Z.J.)

**Keywords:** principal component analysis, space window, time window, damage detection

## Abstract

Long-term structural health monitoring (SHM) has become an important tool to ensure the safety of infrastructures. However, determining methods to extract valuable information from large amounts of data from SHM systems for effective identification of damage still remains a major challenge. This paper provides a novel effective method for structural damage detection by introduction of space and time windows in the traditional principal component analysis (PCA) technique. Numerical results with a planar beam model demonstrate that, due to the presence of space and time windows, the proposed double-window PCA method (DWPCA) has a higher sensitivity for damage identification than the previous method moving PCA (MPCA), which combines only time windows with PCA. Further studies indicate that the developed approach, as compared to the MPCA method, has a higher resolution in localizing damage by space windows and also in quantitative evaluation of damage severity. Finally, a finite-element model of a practical bridge is used to prove that the proposed DWPCA method has greater sensitivity for damage detection than traditional methods and potential for applications in practical engineering.

## 1. Introduction

The safety of infrastructures such as bridges and high-rise buildings is of the utmost concern to the public. During operation, civil structures are subjected to various kinds of external loads, such as traffic, wind, temperature, etc. In fact, these evolving loads may be much more complicated than those considered in the design phase. Therefore, it is of importance to monitor the structural responses, such as strain, displacement, and acceleration with the aim of assessment of their real-time states. Nowadays, long-term structural health monitoring (SHM) systems are widely used to acquire data of structural responses, as well as external loads to monitor the states of civil structures. However, how to process and analyze these data for identifying possible structural changes has been a great challenge [1,2]. In general, structural responses may not change evidently when only a small amount of damage is imparted. Moreover, response variations may be masked by the uncertainties in the structural parameters of practical structures, as well as by the presence of noise. All of these factors result in the raw data being uninformative regarding the occurrence of structural changes, therefore, resulting in the need for feature extraction of measurement data [3]. In order to detect damage effectively, the extracted features are required to be sensitive to damage, while insensitive to parametric uncertainties or noise.

Damage detection methods can be generally classified into two categories, namely model-based [4] and model-free methods [5]. Model-based methods require an accurate finite-element model as well as a model-updating process for damage identification [6]. They have the ability not only to identify the presence and location of damage but also to quantify it in meaningful engineering units. However, computational complexity and model updating of these methods, especially for large-scale structures, have been a challenge in SHM [5]. As an alternative, model-free methods have drawn much attention for the sake that they have been demonstrated applicable to damage identification [7]. These methods utilize time series of measurement data for analysis without the need for geometrical and material information. Due to this reason, they are more inexpensive and efficient compared to model-based methods.

During the past few decades, various kinds of model-free data-interpretation methods for damage detection have been developed, including the autoregressive (AR) model, autoregressive moving average (ARMA) model, autoregressive integrated moving average (ARIMA) model, correlation analysis (CA), instance-based method (IBM), wavelet-based (WB) methods, neural network (NN) model, robust regression algorithm (RRA), principal component analysis (PCA), etc. AR establishes a time-series model to predict future values based on the past measured data. Residual errors or AR parameters are usually used as sensitive features for damage detection [8,9]. ARMA and ARIMA, which are improved methods compared to AR, also take advantage of coefficients as indices for identifying damage [10,11]. CA detects damage through variations of correlation coefficients for measurement datasets since the correlation coefficients will change when damage occurs. This method has been demonstrated to have good performance with regard to identifying and localizing damage [12]. However, it fails to identify damage when the measurement noise is at high levels [13]. IBM computes the minimum distance of a cluster of sensor data (generally for three or four sensors) at each time step [14]. The occurrence of damage is determined if the phase of the minimum distance exceeds a threshold [15]. WB methods are also effective tools for on-line and off-line damage detection [16]. These methods firstly decompose original signals in different time domains and scales. Then, mode shapes, wavelet spectra, wavelet component energy, and the tendency of wavelet coefficients are selected as sensitive features to detect damage [17,18,19,20]. The NN model has been widely utilized to identify anomalous structural behavior by using static and dynamic responses [21,22]. The number of hidden layers, the number of neurons in each layer, the neuron activation function and error criteria should be carefully considered in the NN method [23]. Some investigators also verified that incorporating other methods into a traditional NN model significantly enhances the effectiveness of damage detection [24,25]. As for RRA, it is focused on the correlation between a pair of sensors and construction of a robust regression relationship for measurement data [26]. An anomaly is identified when correlation coefficients exceed threshold bounds. This method has demonstrated the ability to identify and localize damage in simple as well as complex structures [27].

PCA is another popular method used for damage identification in long-term SHM. It exhibits reliable and effective performance in modal analysis, reduced-order modelling, feature extraction, and structural damage detection [28,29,30,31,32]. In addition, it proves to be an effective tool to improve the training efficiency and enhance the classification accuracy for other machine learning algorithms, such as unsupervised learning methods [33,34,35,36,37]. Since the total historical dataset including responses of both healthy and damaged states is involved in the analysis process, PCA is not sensitive to the occurrence of damage in real time in SHM. Moreover, large amounts of historical data may cause computational complexity. Posenato et al. then proposed the moving PCA (MPCA) method to enhance discrimination features between undamaged and damaged structural responses [13,27,38]. This method essentially uses a sliding fixed-size time window for time-series data instead of handling the total historical dataset. An eigenvector time series will be obtained as the time window moves forward. The components of the most important eigenvector are utilized as sensitive features for damage detection. Due to the moving temporal window, MPCA enhances the detection effectiveness compared to that of the traditional PCA method through monitoring the evolution of eigenvector components between undamaged and damage states. In other words, MPCA is used to monitor the components of the eigenvector variance (CEVs) between a healthy state and damaged state for damage identification. It was demonstrated that the sensitivity of MPCA for damage identification was significantly improved compared to other methods such as ARIMA, CWT, RRA and IBM [12,13,39]. However, in the data-interpretation process of both PCA and MPCA, data from all sensors should be used to calculate the eigenvalues and eigenvectors. It makes sense that responses located far from the damaged area are insensitive to damage. In other words, part of the data includes information insensitive to damage, consequently reducing the sensitivity regarding damage detection. If a space window is applied to exclude the data from those sensors located far from the damage, it is possible to improve the damage detectability. As a consequence, if both space and time windows are applied in the traditional PCA method, this is expected to further improve the damage detectability. In fact, Posenato et al. have also proposed a sensor clustering overlapping algorithm for MPCA when there exists a large number of sensors [13]. The clustering process is essential to implement space windows for the installed sensors. However, the authors aimed to deal with measurements from fewer sensors for computational efficiency. They did not carry out further investigation on the damage detectability.

According to the above discussion, both PCA and MPCA methods use all sensors for analysis and may decrease the detection performance. This paper will provide a double-window PCA (DWPCA) method for structural damage identification. The primary idea is to combine space and time windows with the traditional PCA method. It is found that discrimination of the eigenvectors between damaged and healthy states is enhanced due to the introduction of space and time windows. Numerical results show that the proposed method, in contrast with MPCA, improves the sensitivity for damage identification and is also quicker to detect damage after its occurrence. Further investigations indicate that the novel approach exhibits a better performance regarding damage localization and quantitative evaluation. Finally, the proposed DWPCA is shown to be robust in the presence of noise and shows potential for applications in practical engineering.

This paper is organized as follows: Section 2 describes PCA, MPCA and the proposed DWPCA method. Section 3 presents a detailed description of the planar beam model for simulations, as well as the methodology to determine the space window. In Section 4, comparative studies with MPCA are conducted to verify the advantages of the proposed method. In Section 5, application of the proposed DWPCA to a full-scale structure is presented. In Section 6, valuable conclusions are drawn according to the numerical results.

## 2. The Proposed Double-Window Principal Component Analysis Method

In the following, the descriptions of PCA, MPCA, and the proposed DWPCA method will be presented in sequence. It should be noted that MPCA introduces a moving time window in the traditional PCA method, while the proposed DWPCA introduces both space and time windows.

### 2.1. PCA

PCA is a useful tool for reducing data dimensionality while retaining essential information for manipulated datasets. The main objective is to transform original data to a smaller set of uncorrelated variables [40]. For damage detection, PCA can be used to eliminate noise and simultaneously derive damage-sensitive features such as eigenvectors. The data-processing steps of PCA are detailed as below. The first step of PCA is the construction of a matrix, U(t), that contains the time histories of all measured data:

(1) $$U(t)=\left[\begin{array}{cccc} u_{1}(t_{1}) & u_{2}(t_{1}) & \cdots & u_{M}(t_{1})\\ u_{1}(t_{2}) & u_{2}(t_{2}) & \cdots & u_{M}(t_{2})\\ \vdots & \vdots & \ddots & \vdots \\ u_{1}(t_{N}) & u_{2}(t_{N}) & \cdots & u_{M}(t_{N}) \end{array}\right],$$

where *t* represents time, $u_{i}\left(i=1,\;2,\;\cdots ,\;M\right)$ denotes the response from the *i*-th sensor installed in the monitored structure, *M* is the total sensor number, $t_{j}\left(j=1,\;2,\;\cdots ,\;N\right)$ denotes the *j*-th time step of measurements, and *N* is the total number of time observations during monitoring. Note that the data of each column are the time series of measurement events from each individual sensor.

Subsequently, time series of each column or each sensor should be normalized by subtracting the mean value given by:

(2) $${\bar{u}}_{i}=\frac{1}{N}\sum_{j=1}^{N}u_{i}(t_{j}).$$

The normalized matrix can then be written as:

(3) $$U^{\prime }(t)=\left[\begin{array}{cccc} u_{1}(t_{1})-{\bar{u}}_{1} & u_{2}(t_{1})-{\bar{u}}_{2} & \cdots & u_{M}(t_{1})-{\bar{u}}_{M}\\ u_{1}(t_{2})-{\bar{u}}_{1} & u_{2}(t_{2})-{\bar{u}}_{2} & \cdots & u_{M}(t_{2})-{\bar{u}}_{M}\\ \vdots & \vdots & \ddots & \vdots \\ u_{1}(t_{N})-{\bar{u}}_{1} & u_{2}(t_{N})-{\bar{u}}_{2} & \cdots & u_{M}(t_{N})-{\bar{u}}_{M} \end{array}\right].$$

The next step is to construct the M×M covariance matrix, which is defined as:

(4) $$C=\frac{1}{M}{U^{\prime }}^{T}U^{\prime }.$$

Finally, the eigenvalue $\lambda_{i}$ and the corresponding eigenvector $\psi_{i}$ of the covariance matrix can be obtained by solving the following equation:

(5) $$(C-\lambda_{i}I)\psi_{i}=0,$$

where I denotes the M×M identity matrix, $\psi_{i}={\left[\begin{array}{cccc} \psi_{i,1} & \psi_{i,2} & \cdots & \psi_{i,M} \end{array}\right]}^{T}$ in which $\psi_{i,j}\left(j=1,\;2,\;\cdots ,\;M\right)$ is the component corresponding to the jth sensor.

Generally, one would sort the eigenvalues into decreasing order, namely $\lambda_{1}>\lambda_{2}>\cdots >\lambda_{M}$. Then, the first eigenvector $\psi_{1}$ related to $\lambda_{1}$ contains the largest variance and thereby retains essential information for the original matrix U. In fact, most of the variance is contained in the first few principal components, while the remaining less important components involve the measurement of noise. For this reason, the first few eigenvectors are always used as sensitive features to detect and localize damage. It can be seen that neither a space window nor time window is applied in PCA. The total historical dataset including responses of healthy and damaged states is used for analysis, thereby leading to low damage detectability.

### 2.2. MPCA

MPCA is an improved method based on PCA which involves applying a moving time window of fixed size. Only the time series of observations inside the moving time window are used to construct the covariance matrix for the derivation of eigenvalues and eigenvectors. Previous studies have proven that the introduction of the moving time window enhances the discrimination between features of undamaged and damaged structures, and thereby renders better performance for damage detection [14,26]. Additionally, the sensitivity of MPCA for damage identification has proven to be significantly improved as compared with other methods such as PCA, ARIMA, DWT, RRA and IBM [12,13,39]. The proper choice of the window size *T* is also important in the first step. If the response time series have periodic characteristics, the temporal window size should be equivalent to the longest period. Once the time window size or the number of consecutive measurements for each sensor inside the window is fixed, the matrix **U** in Equation (1) at the *k*-th time step can be rewritten as:

(6) $$U(k)=\left[\begin{array}{cccc} u_{1}(t_{k}) & u_{2}(t_{k}) & \cdots & u_{M}(t_{k})\\ u_{1}(t_{k+1}) & u_{2}(t_{k+1}) & \cdots & u_{M}(t_{k+1})\\ \cdots & \cdots & \ddots & \cdots \\ u_{1}(t_{k+T-1}) & u_{2}(t_{k+T-1}) & \cdots & u_{M}(t_{k+T-1}) \end{array}\right],$$

where k=1, 2, ⋯, N−T+1. Note that the mean value of each column of U(k) at the *k*-th time step would become,

(7) $${\bar{u}}_{i}(k)=\frac{1}{T}\sum_{j=k}^{k+T-1}u_{i}(t_{j}).$$

Next, repeating the steps of PCA, one is able to obtain the eigenvalue $\lambda_{i}(k)$ and eigenvector $\psi_{i}(k)$. It should be noted that $\lambda_{i}(k)$ and $\psi_{i}(k)$ are time series and vary with the time step. 

During continuous monitoring, responses are divided into two phases: training and monitoring phases. In the training phase, the structure is assumed to behave normally (no damage). Then, eigenvector variance between the training phase and the monitoring phase at the kth time step can be determined by the following equation:

(8) $$\Delta \psi_{i}(k)=\psi_{i}(k)-{\bar{\psi }}_{i},$$

where ${\bar{\psi }}_{i}$ denotes the mean value of the ith eigenvector in training phase, while $\Delta \psi_{i}(k)$ represents the eigenvector variance between $\psi_{i}(k)$ and ${\bar{\psi }}_{i}$ at the kth time step of the monitoring phase, and $\Delta \psi_{i}\left(k\right)=[\Delta \psi_{i,1}\left(k\right)\;\Delta \psi_{i,2}\left(k\right)\;\cdots \;\Delta \psi_{i,M}\left(k\right)]{}^{T}$ where $\Delta \psi_{i,j}\left(k\right)$ is the component of the eigenvector variance (CEV) corresponding to the jth sensor. It should be noted that $\Delta \psi_{i,j}\left(k\right)$ is generally utilized as the feature for anomaly detection in MPCA. CEV $\Delta \psi_{i,j}\left(k\right)$ by MPCA can be expressed in terms of eigenvector components as follows:

(9) $$\Delta \psi_{i,j}(k)=\psi_{i,j}(k)-{\bar{\psi }}_{i,j},$$

where ${\bar{\psi }}_{i,j}$ denotes the mean value of the eigenvector component in a healthy state, and $\Delta \psi_{i,j}(k)$ can be considered as the variation between $\psi_{i,j}(k)$ in monitoring phase and ${\bar{\psi }}_{i,j}$ in healthy state. If $\Delta \psi_{i,j}(k)$ exceeds a threshold, alarm will be flagged.

When damage occurs, structural responses may change, consequently causing variations in eigenvectors and CEVs. Thus, one may follow the $\Delta \psi_{i,j}(k)$ over time to examine whether damage exists. As indicated in Equation (6), MPCA uses only the latest *T* observations instead of the whole time series. Once damage occurs, fewer data that are irrelevant to the damage, as compared with PCA, are considered for the calculation, resulting in a better sensitivity for damage detection. However, MPCA generally takes into account responses from all sensors. Some of the sensors may be insensitive to damage located at a certain position. If a space window is used to group sensitive sensors, it is possible to enhance damage detectability.

### 2.3. The Proposed DWPCA

When damage occurs, data from sensors close to damage location change significantly while data from sensors away from damage may be unchanged. Hence, a novel DWPCA method is proposed herein to combine space and time windows with PCA. It can also be treated as an improved method for MPCA by the introduction of a space window. The application of the space window, in the aim of enhancing damage detectability, is to group sensors sensitive to damage and to exclude those that are insensitive. The key step for the choice of the space window is to determine the damage-sensitive area (DSA), where measurement data change significantly when damage occurs. It should be noted that the DSA varies with damage location as well as damage level. For example, the damage location commonly decides the position of the DSA and a high damage level reasonably leads to a large DSA.

For the space window, a criterion as shown below is used for determination of the damage-sensitive sensors which fall within the DSA:

(10) $$\left|\left[u_{i}^{d}(t)-u_{i}^{h}(t)\right]/u_{i}^{h}(t)\right|\geq \eta ,$$

where the superscripts d and h denote damaged and healthy states, respectively. For each measurement time step, $u_{i}^{d}(t)$ represents the data from the *i*-th sensor in a damaged case, and $u_{i}^{h}(t)$ represents the response in a healthy state. η is the lowest limit of a detectable relative change in response. Note that $\Delta u_{i}^{}(t)=u_{i}^{d}(t)-u_{i}^{h}(t)$ represents the variation of response under a damage condition. If $\left|\Delta u_{i}^{}(t)/u_{i}^{h}(t)\right|$ is lower than the sensor sensitivity, it is impossible to detect the damage. Therefore, η should be chosen as the sensor sensitivity. It should be noted that Equation (10) only applies to responses which are sensitive to local damage. And in this paper, strains are used in the analysis. However, Equation (10) may not be applicable to vibration monitoring, due to the fact that vibration responses are integral structural effects and may not be sensitive to local damage.

A sensor is defined as damage-sensitive if the responses it acquires in the case of damage satisfy the following formula:

(11) $$n_{d}/n\geq p_{0},$$

where n represents the total number of observations over time, $n_{d}$ represents the total number of observations satisfying Equation (10), $p_{0}$ is a given constant parameter that determines the lowest possibility to define a damage-sensitive sensor. In fact, it is difficult to determine the exact value for $p_{0}$ because it depends on the specific structures. In general, an approximate range for $p_{0}$ can be given as from 50% to 100%. This means if more than half of all observations at a certain scenario satisfy Equation (10), then a sensor can be treated as damage-sensitive. Once an accurate FE model is given, an accurate method for determining DSA according to Equation (11) can be provided based on numerical simulations of various damaged cases (various combinations of damage locations and severities are considered). Consequently, the space window can be defined as the set of sensors installed in DSA. However, it is not possible to provide an accurate method to determine the DSA without an accurate FE model, because the determination of DSA depends on specific structures including materials, types of structures and boundary conditions. In such case, only empirical experience is available to determine the DSA and the space window. For example, one can use diverse space windows of which each involves several neighboring sensors. Because damage in general has more effects on those sensors which are nearby, the space window that is nearest to the location of the damage is most likely to group the sensors that are more sensitive to damage.

The space window is presented in the form of $\left[\begin{array}{cccc} i_{1} & i_{2} & \cdots & i_{S} \end{array}\right]$, where $i_{1},\;i_{2},\;\cdots ,\;i_{S}$ are the sensor numbers, and S represents the total number of sensors in the space window, in other words, within the DSA. For example, $\left[\begin{array}{cccc} 1 & 3 & 5 & 7 \end{array}\right]$ means Sensor 1, Sensor 3, Sensor 5, and Sensor 7 are grouped inside the space window. Once the window is determined, one can conduct PCA with a moving time window for measurement values to detect damage. In consideration of space and time windows, Equation (6) can be rewritten as:

(12) $$U(k)=\left[\begin{array}{cccc} u_{i_{1}}(t_{k}) & u_{i_{2}}(t_{k}) & \cdots & u_{i_{S}}(t_{k})\\ u_{i_{1}}(t_{k+1}) & u_{i_{2}}(t_{k+1}) & \cdots & u_{i_{S}}(t_{k+1})\\ \cdots & \cdots & \cdots & \cdots \\ u_{i_{1}}(t_{k+T-1}) & u_{i_{2}}(t_{k+T-1})) & \cdots & u_{i_{S}}(t_{k+T-1}) \end{array}\right].$$

Then, repeating the steps of PCA, one is able to obtain the time-variant eigenvector $\psi_{i}(k)={\left[\begin{array}{cccc} \psi_{i,1} & \psi_{i,2} & \cdots & \psi_{i,S} \end{array}\right]}^{T}$. Similarly, with Section 2.2, herein $\Delta \psi_{i,j}\left(k\right)$ by DWPCA in a spatial window can be obtained from Equation (9). By following the CEV $\Delta \psi_{i,j}\left(k\right)$ at each time step, damage can be detected if $\Delta \psi_{i,j}\left(k\right)$ exceeds a certain threshold value. Meanwhile, it is possible to localize damage through the space window by observing rapidly changing components. It can be seen in Equation (12) that only sensitive responses are considered in the analysis for DWPCA. Thus, it is expected to improve the performance of damage identification.

## 3. Validation of DWPCA with a Planar Beam

### 3.1. Model for Simulation

**Figure 1:**
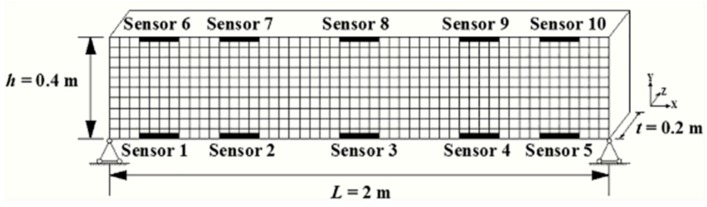
Finite element model of a simply supported beam for simulations with ten strain sensors installed.

To evaluate the effectiveness and efficiency of the proposed DWPCA method, large datasets including responses from a structure under various damaged scenarios are needed. In practice, it is not possible to acquire such datasets from a real civil structure because intentionally imparting damage to the structure is not allowed for safety reasons. As a result, this study adopts a finite element (FE) model of a planar beam established by ANSYS for calculations to obtain necessary datasets, as shown in Figure 1. In the simulations, strain responses of the FE model under seasonal temperature variations are computed under different damaged scenarios. Simulations for various damage severities at different locations are achieved by exerting certain stiffness reductions in certain finite elements of the model.

**Figure 2:**
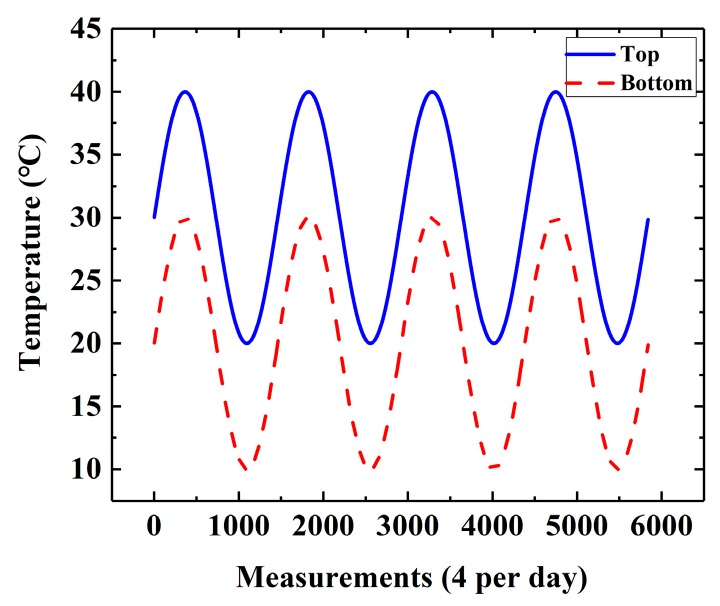
Evolution of temperatures at the top and bottom of the beam over four years.

The planar beam in Figure 1 is assumed to be 2.0 m in length (L) with a rectangular cross-section of 0.4 m in height (h) and 0.2 m in width (t). The FE model is evenly discretized into 500 quadrilateral elements with 50 elements for each row in the x-direction (beam length) and 10 elements for each column in the y-direction (beam height). Note that each element of the meshes has a length of 0.04 m and a height of 0.04 m. It is also assumed that the beam is composed of concrete with a Young’s modulus of 34.5 GPa, a Poisson’s ratio of 0.2, and a thermal expansion coefficient of $1\times 10^{-5}{/}^{{}^{\circ}}C$. For the thermal loads, seasonal temperature variations are applied on the bottom and top surfaces. The temperature on the bottom surface is set to be $T_{b}=20+10\sin(\pi t/730)({}^{{}^{\circ}}C)$, while that on the top surface is $T_{t}=T_{b}+10({}^{{}^{\circ}}C)$. Note that the sinusoidal function in $T_{b}$ has a period of one year, which is consistent with the period of seasonal temperature variations. In addition, a linear temperature distribution along the beam height is taken into consideration. During simulations, equivalent forces caused by temperature variations are exerted on the nodes of each finite element to obtain strain responses. In this paper, thermal excitations of four years are applied. Figure 2 illustrates the evolution of temperatures at the top and bottom of the beam over four years. 

Additionally, a virtual SHM system is installed in the beam structure. As shown in Figure 1, the system is assumed to be composed of ten strain sensors, of which five are installed on the top surface and the other five on the bottom. For each damaged scenario, strain histories are computed for four years with four measurements per day, i.e., 5840 measurement events in total for each sensor. In addition, permanent damage is introduced at the beginning of the third year (after 2920 measurements) by stiffness reductions at certain finite elements in the model.

**Figure 3:**
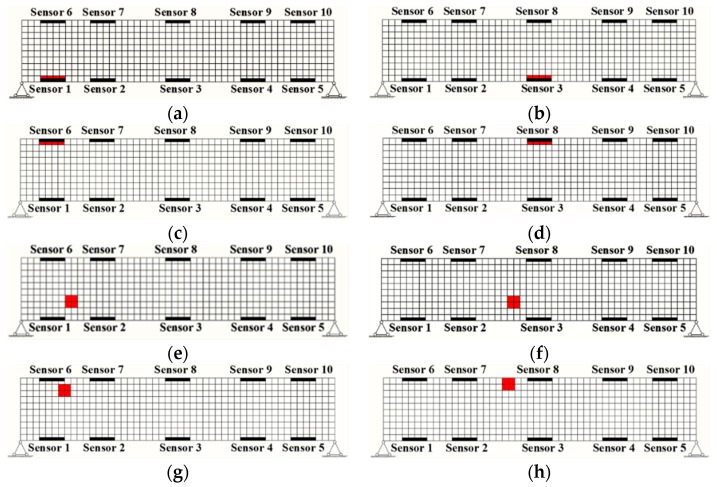
Damaged scenarios for the evaluation of damage detection algorithms: (**a**) damage in four finite elements at Sensor 1; (**b**) damage in four finite elements at Sensor 3; (**c**) damage in four finite elements at Sensor 6; (**d**) damage in four finite elements at Sensor 8; (**e**) damage in four finite elements near Sensor 1; (**f**) damage in four finite elements near Sensor 3; (**g**) damage in four finite elements near Sensor 6; (**h**) damage in four finite elements near Sensor 8.

**Figure 4:**
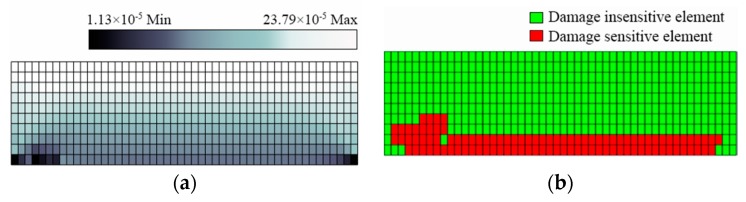
Simulation results for Scenario A with a stiffness reduction of 80%: (**a**) contour for strain variation; (**b**) damage-sensitive area indicated by finite elements in red.

**Figure 5:**
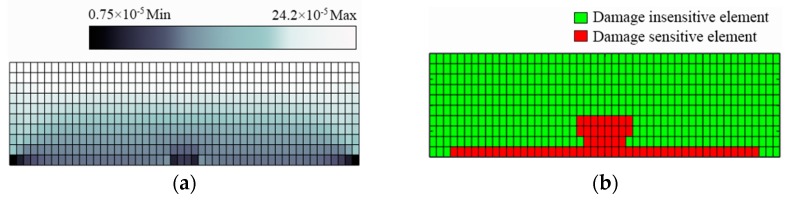
Simulation results for Scenario B with a stiffness reduction of 80%: (**a**) contour for strain variation; (**b**) damage-sensitive area indicated by finite elements in red.

**Figure 6:**
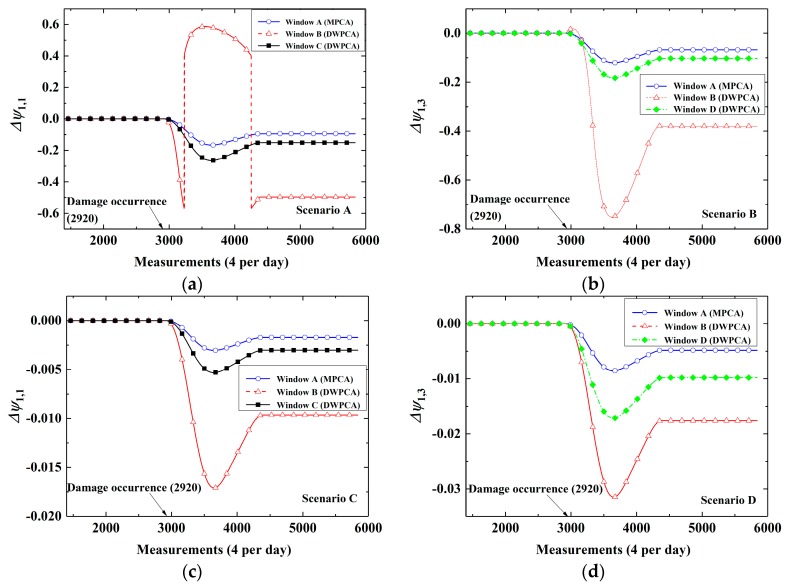
Evolution of CEVs for different space windows: (**a**) Scenario A; (**b**) Scenario B; (**c**) Scenario C; (**d**) Scenario D.

**Figure 7:**
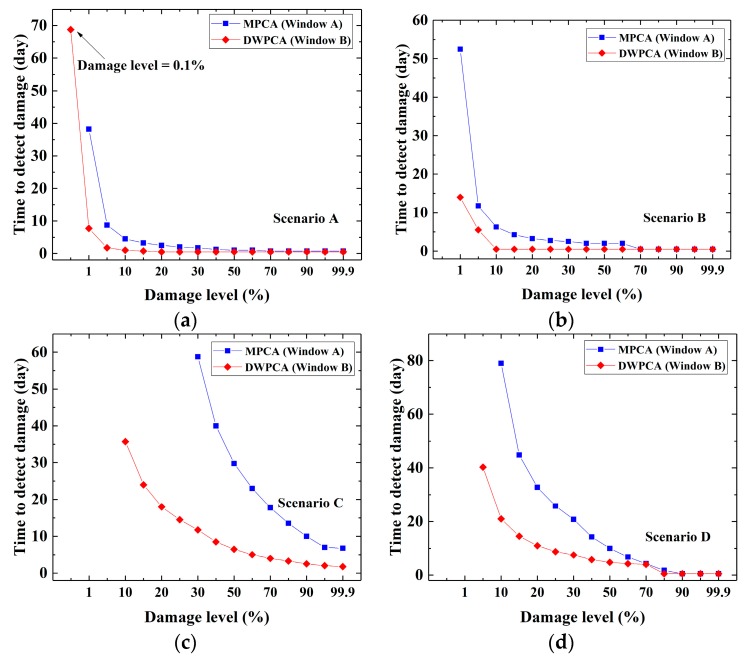
Time to detect damage after damage occurrence with respect to the damage level: (**a**) Scenario A; (**b**) Scenario B; (**c**) Scenario C; (**d**) Scenario D.

**Figure 8:**
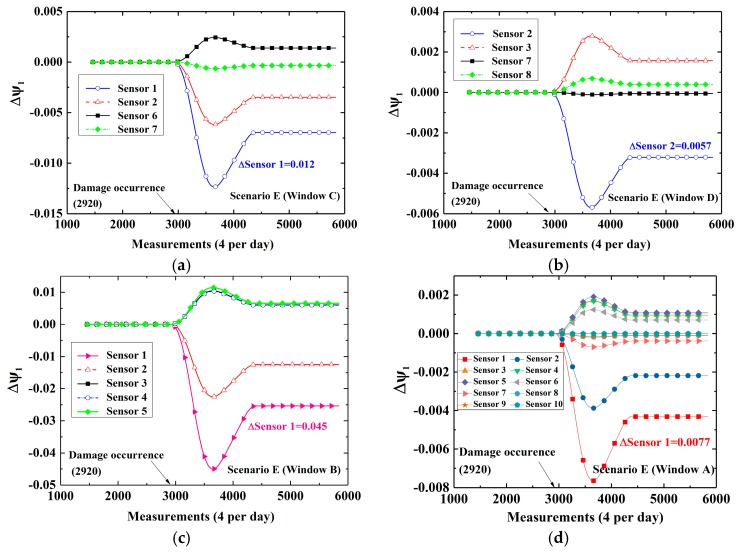
Time-variant CEVs with different space windows for Scenario E: (**a**) Window C; (**b**) Window D; (**c**) Window B; (**d**) Window A.

In this paper, the following damaged scenarios, as illustrated in Figure 3, are considered for comparative studies between MPCA and the proposed DWPCA:

(1). Scenario A: Damage in four finite elements at Sensor 1 as shown in Figure 3a;
(2). Scenario B: Damage in four finite elements at Sensor 3 as shown in Figure 3b;
(3). Scenario C: Damage in four finite elements at Sensor 6 as shown in Figure 3c;
(4). Scenario D: Damage in four finite elements at Sensor 8 as shown in Figure 3d;
(5). Scenario E: Damage in four finite elements near Sensor 1 as shown in Figure 3e;
(6). Scenario F: Damage in four finite elements near Sensor 3 as shown in Figure 3f;
(7). Scenario G: Damage in four finite elements near Sensor 6 as shown in Figure 3g;
(8). Scenario H: Damage in four finite elements near Sensor 8 as shown in Figure 3h.

### 3.2. Determination of the DSA

To determine the DSA in this study, the value of the parameter η is chosen as 5% because the sensor sensitivity is assumed to be 5% in the simulation. As for $p_{0}$, it is chosen as 60%, because it is observed that if more than 60% of all observations in a certain scenario satisfy Equation (10), the sensor is found to be damage-sensitive according to the simulation results. Figure 4 and Figure 5 illustrate the calculated strain variation, as well as the DSA, for Scenario A and Scenario B with a stiffness reduction of 80%, respectively. It is seen that damage-sensitive elements are indeed more likely to lie in the vicinity of the damage location. However, as shown in Figure 4b and Figure 5b, some finite elements are sensitive to damage even though they are located far from the damage location. As a result, the determination of the DSA should not be directly based on damage location. Numerical simulations can help this problem.

In consideration of the simulation results for different damaged scenarios, as well as the symmetry of the FE model in Figure 1, the following four listed windows are used for comparative studies between MPCA and the proposed DWPCA:

(1). Window A involving all the sensors: $\left[\begin{array}{cccccccccc} 1 & 2 & 3 & 4 & 5 & 6 & 7 & 8 & 9 & 10 \end{array}\right]$;
(2). Window B involving all the sensors at the bottom: $\left[\begin{array}{ccccc} 1 & 2 & 3 & 4 & 5 \end{array}\right]$;
(3). Window C: $\left[\begin{array}{cccc} 1 & 2 & 6 & 7 \end{array}\right]$;
(4). Window D: $\left[\begin{array}{cccc} 2 & 3 & 7 & 8 \end{array}\right]$.

It should be noted that Window A is usually implemented in PCA and MPCA. If other windows are applied, the algorithm will belong to the DWPCA method. In the following calculations, the time window size for both MPCA and DWPCA is equal to one period of the thermal loads, namely one year (1460 measurement events). The first principal component $\psi_{1}$ is considered as the sensitive feature for damage detection because most of the variance is contained in it.

## 4. Results and Discussion

In this section, comparative studies between DWPCA and MPCA in previous studies for damage detection will be carried out on detection sensitivity, damage localization, and severity evaluation. Noise immunity of the proposed features will also be investigated. To begin with, the effects of the following two features on damage identification performance are investigated:

(1). CEV by MPCA: $\Delta \psi_{i,j}^{M}$;
(2). CEV by DWPCA: $\Delta \psi_{i,j}^{DW}$.

Note that Window A is usually implemented in MPCA. Other windows including Window B to Window D are implemented in the DWPCA method.

### 4.1. Sensitivity for Damage Detection

To begin with, the effects of different windows on the time series CEVs are investigated. In the simulations, four scenarios (A, B, C, and D) and four windows (A, B, C, and D) are considered for a comparative study. In addition, a permanent stiffness reduction of 40% was introduced for the corresponding finite elements at the beginning of the third year. Note that if Window A is used, the method belongs to MPCA because all installed sensors are taken into account. As shown in Figure 6, the time-variant CEVs of different scenarios and space windows are simulated. It can be seen that before the damage occurs, CEVs are stable for all cases. However, there exists a shift for the values after damage occurrence at all scenarios. In the unstable stage between the 2920th and 4380th measurements, strains within the moving time window involve responses from both undamaged and damaged states. As the time window moves forward, the corresponding CEVs will become stable again after 4380 measurement events. This results from the fact that all strain time series within the time window were obtained from the damaged structure after 4380 measurements. A closer look at Figure 6 indicates that a more significant change is observed for the proposed DWPCA method with Windows B, C, and D in contrast to MPCA with Window A. It is interesting to find that among the used space windows, Window B, that contains all the sensors installed at the bottom as shown in Figure 1, renders a more rapid and evident change in CEVs for the considered damaged scenarios as compared with the healthy state. The result can be explained by the fact that, as illustrated by the simulation results in Figure 4 and Figure 5, damage has a greater influence on the responses at the bottom. From a mechanical point of view, the sensors at the bottom are near the constraint boundaries and any variations in the structure may lead to a more significant change in their responses. Additionally, one can see from Figure 6 that damage at the bottom has more influence on structural responses and CEVs than damage at the top, indicating that it is easier to detect damage at the bottom. In a word, the proposed DWPCA uses a space window to exclude sensors outside the DSA, thereby leading to an enhanced sensitivity for damage detection as compared with MPCA.

In order to conduct a comparative study on the detection resolution and the time to detect damage after damage occurrence between MPCA and the proposed DWPCA, a range of damage severities are considered for simulations. Detection resolution is defined as the damage level that induces a minimum detectable relative variation of CEVs in comparison with those in a healthy state. In this paper, the minimum detectable relative CEV is chosen as the value when the variation rate of the corresponding CEV with respect to time is equal to 0.67 με/h. The time to detect damage is the period from the moment the damage occurs to that when damage is detected. Figure 7 presents the time to detect damage after damage occurrence with respect to damage levels ranging from the minimum detectable level of each method to a maximum level of 99.9% in Scenarios A, B, C and D. For DWPCA, the space window of Window B is considered in the simulations. As expected, higher damage levels at any scenario result in a shorter detection time of damage for both methods. However, as the damage level is lower than 70% for the considered scenarios, DWPCA shows a shorter time to detect damage as compared with MPCA. Furthermore, this advantage of DWPCA is increasingly evident as the damage level becomes smaller. Note that, usually, structural damage emerges is initially small and gradually evolves to larger damage. The use of a space window means that DWPCA possesses a superior capability in the early detection of damage and timely alarms in contrast to MPCA.

Table 1 presents the detection resolution of both methods according to the simulation results in Figure 7. It can be seen that DWPCA has a better detection resolution than MPCA for all scenarios, except for Scenario B, in which the same resolution is observed for both methods. In fact, the damage in Scenario B is located at the bottom of the mid-span and has significant effects on the structural responses. Hence, MPCA exhibits a comparative detection resolution even though a space window is not applied. For other cases, the detection resolution of DWPCA is commonly better than that of MPCA, owing to the fact that the space window in DWPCA excludes sensors that are not sensitive to damage. Further examination of Table 1 shows damage located at the bottom (Scenarios A and B) is easier to detect than that located at the top (Scenario C and D) because damage at the bottom, generally, has more significant effects on the structural responses. In addition, DWPCA has the best detection resolution of 0.1% in Scenario A, in which the damage is located at the bottom near the constraint boundary, while it has the worst detection resolution of 10% for Scenario C, in which the damage is located at the top near the sides of the beam. Table 2 presents the time to detect damage at a minimum common level for both MPCA and DWPCA in different scenarios. It is clear that DWPCA detects damage much earlier than MPCA. DWPCA takes about 7 to 21 days to detect damage after its occurrence, while MPCA needs about 38 to 80 days. Note that in Scenario B, DWPCA detects damage more rapidly than MPCA although MPCA has the same resolution as DWPCA. In addition, it is also apparent that damage at the bottom (Scenarios A and B) is easier and quicker to detect than that at the top (Scenarios C and D). In summary, it is demonstrated that damage detectability of the proposed DWPCA is improved as compared with MPCA due to the application of a space window which groups sensors within the DSA and excludes those insensitive to damage.

### 4.2. Damage Localization

In Section 4.1, DWPCA has been demonstrated as a more effective tool to identify damage than MPCA. In this subsection, a methodology for damage localization will be put forward by tracking the time-variant CEVs based on the proposed DWPCA. Four Scenarios (E, F, G, and H) in Figure 3 and a stiffness reduction of 40% are considered.

**Figure 9:**
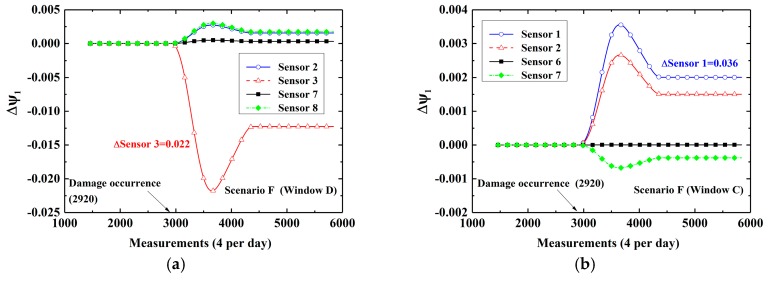

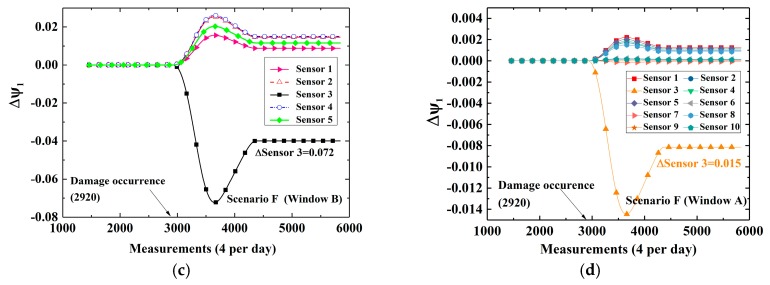
Time-variant CEVs with different space windows for Scenario F: (**a**) Window D; (**b**) Window C; (**c**) Window B (**d**) Window A.

**Figure 10:**
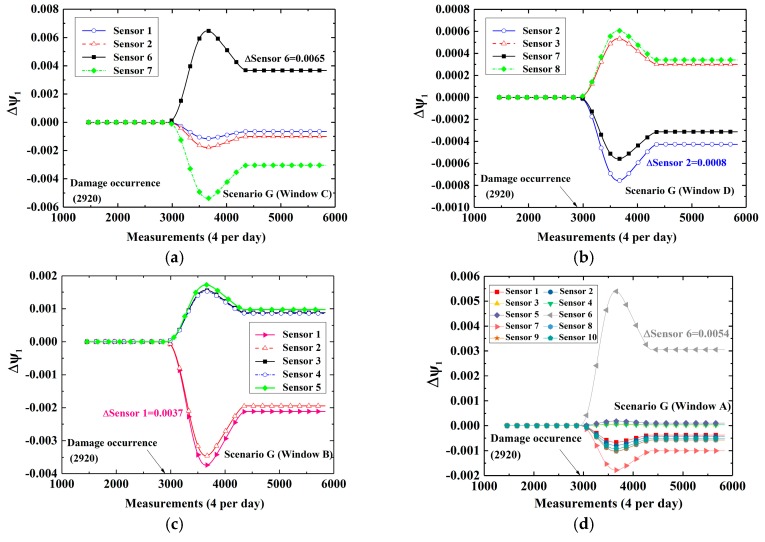
Time-variant CEVs with different space windows for Scenario G: (**a**) Window C; (**b**) Window D; (**c**) Window B; (**d**) Window A.

**Figure 11:**
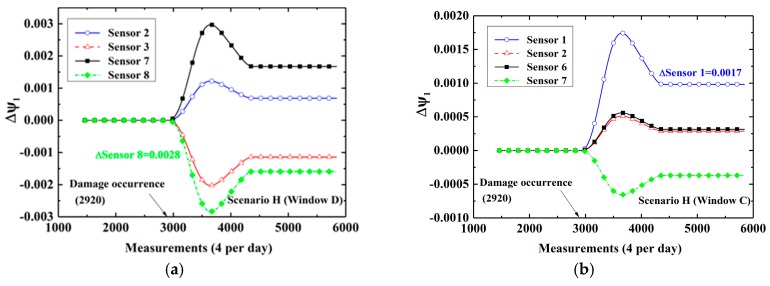

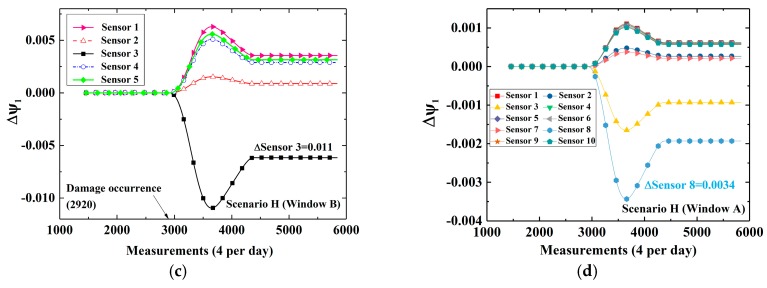
Time-variant CEVs with different space windows for Scenario H: (**a**) Window D; (**b**) Window C; (**c**) Window B; (**d**) Window A.

At first, cases in which damage is located at the bottom (Scenarios E and F) are considered. Figure 8a–c show time-variant CEVs computed by DWPCA with different space windows for Scenario E, in which the damage is located at the bottom between Sensor 1 and Sensor 2. For Window C, as shown in Figure 8a, the CEVs corresponding to Sensor 1 and Sensor 2 show evident shifts after the occurrence of damage as compared with those corresponding to Sensor 6 and Sensor 7. For Window D, shown in Figure 8b, only the CEV corresponding to Sensor 2 has a relatively evident change owing to the fact that Sensor 2 is the sensor that is located closest to the damage in the space window. As for Window B, the CEV corresponding to Sensor 1 exhibits the most evident variation as compared with other components. For Window A, as shown as Figure 8d, the CEV corresponding to Sensor 1 by MPCA is smaller than that by DWPCA with Window C in Figure 8a or Window B in Figure 8c. It demonstrates that the CEV corresponding to Sensor 1 computed by DWPCA is larger than that obtained by MPCA in Scenario E. This proves that DWPCA is more sensitive for damage localization than MPCA. In addition, one can infer from Figure 8 that damage is located close to Sensor 1 because the variation of the corresponding CEV in various windows is the most notable. For Scenario F, in which the damage is located close to Sensor 3, similarly, the CEV related to Sensor 3 shows a significant change, as presented in Figure 9, especially for both Windows B and D as compared with Window A. Subsequently, we consider cases in which damage is located at the top (Scenarios G and H). The simulation results are shown in Figure 10 for Scenario G and in Figure 11 for Scenario H, respectively. For Scenario G, as expected, the CEV related to Sensor 6 is the most evident because the damage is in the vicinity of Sensor 6. As for Scenario H, the CEV related to Sensor 8 displays an evident shift. From Figure 8 to Figure 11, we can see that damage at the bottom has more significant effects on the corresponding CEV as compared with that at the top. Furthermore, if the damage is located at the bottom, Window B shows a better performance for damage localization because a larger variation is observed for the CEV. However, if the damage is located at the top, Window C or D is preferred. In conclusion, it is seen that DWPCA can be used to localize damage with the aid of various space windows and shows a better performance for damage localization as compared with MPCA.

### 4.3. Quantitative Evaluation of Damage

**Figure 12:**
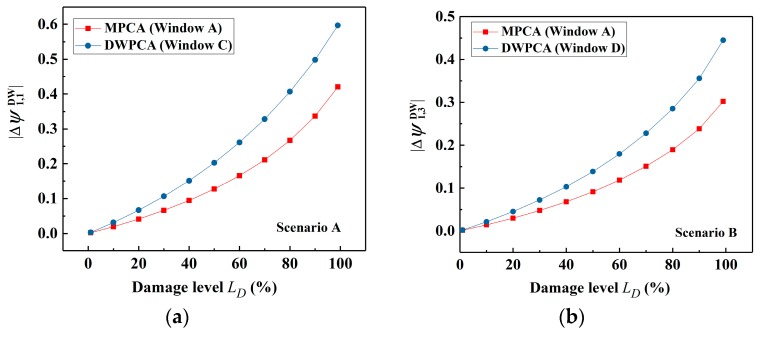
Variation of the absolute value of the CEVs with damage level by MPCA and DWPCA: (**a**) Scenario A; (**b**) Scenario B.

Based on the discussion above, a further investigation to provide a quantitative evaluation of the damage using DWPCA is presented in this section. The relationship between the damage level and stable absolute value of CEV after damage occurrence for a range of damage severities from 0.1% to 99.9% in Scenarios A and B is presented in Figure 12. It can be seen from Figure 12a that the CEV corresponding to Sensor 1 has a monotonically ascending trend as the damage level increases for both MPCA with Window A and the proposed DWPCA with Window C. However, the corresponding CEV for DWPCA with Window C varies more dramatically as a function of the damage level than that of MPCA. A more evident discrimination between data from damaged and undamaged states is observed for the proposed method. Thus, DWPCA has a higher sensitivity for quantitative evaluation of damage as compared with MPCA. According to the simulation results of Scenario A in Figure 12a, the damage level $L_{D}$ can be quantitatively evaluated in terms of $\left|\Delta \psi_{1.1}\right|$ by DWPCA with Window C, as indicated by:

(13) $$L_{D}=0.714\ln\left(\left|\Delta \psi_{1.1}^{DW}\right|+0.190\right)+1.175$$

For Scenario B, as illustrated in Figure 12b, the related CEV also increases with an increase in damage level for MPCA with Window A or DWPCA with Window D. Similarly, the variation of CEV related to Sensor 3 by DWPCA is larger as compared with MPCA. 

Thus, the proposed DWPCA has a higher sensitivity for damage evaluation. For DWPCA with Window D, as presented in Figure 12b, the damage level $L_{D}$ can be obtained from the calculated CEV $\Delta \psi_{1.3}$ with the use of the following equation:

(14) $$L_{D}=0.588\ln\left(\left|\Delta \psi_{1.3}^{DW}\right|+0.090\right)+1.385$$

It should be noted that the relationship between the CEV and damage level is obtained by curve fitting. This methodology for quantitative evaluation requires calibration or training with an accurate FE model.

### 4.4. Noise Immunity

**Figure 13:**
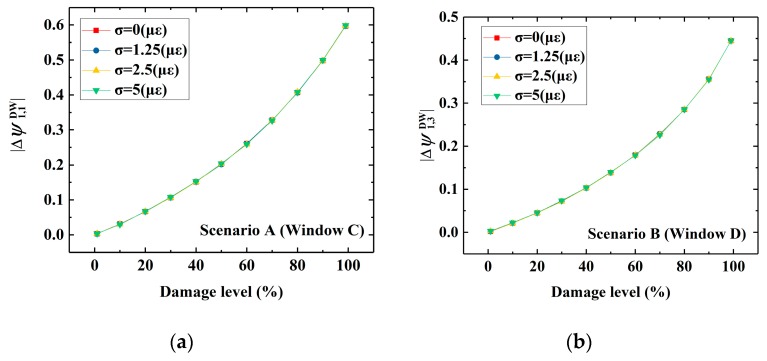
Variation of the absolute value of the CEVs with damage level by DWPCA in the presence of different intensities of noise: (**a**) Scenario A; (**b**) Scenario B.

In practice, noise caused by external environmental factors or systematic errors in SHM is inevitable. Consequently, data from SHM systems involve noise and may render damage identification methods ineffective. As a result, noise immunity of the proposed DWPCA method should be investigated. Based on the measured strain data in a large-scale bridge from the literature [39], the standard deviation of noise is considered to be from 1.25 με to 5 με. Note that strain responses in the simulations are approximately 150 με. Thus, the noise level ranges from 0.8% to 3.3%. The relationship between CEV and damage level in Scenarios A and B, in which different intensities of noise are present is presented in Figure 13. It can be seen that noise has little influence on the relationship between the CEV absolute value and the damage level in DWPCA. This is due to the favorable de-noising characteristic of PCA. In a word, the proposed DWPCA method in this study has considerably good noise immunity and shows potential for applications in practical engineering.

## 5. Application to a Full-Scale Structure

**Figure 14:**
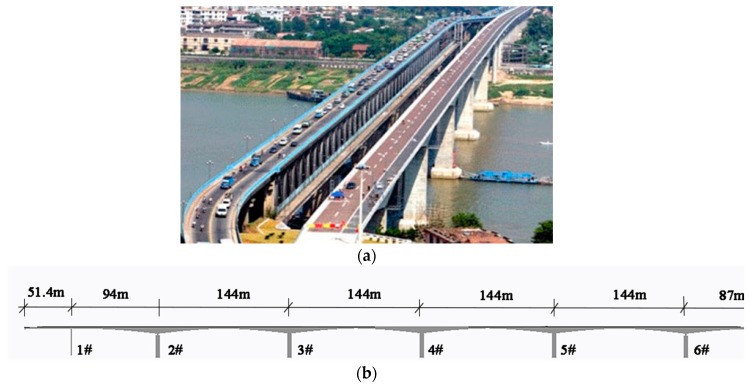
Xijiang Bridge: (**a**) photograph of the bridge; (**b**) schematic diagram of the structure.

Based on the validation for DWPCA with a planar beam in Section 3 and Section 4, in this section, further investigation of the performance of DWPCA for a large-scale structure will be carried out to demonstrate its applicability for practical engineering purposes. The full-scale FE model will be based on the Xijiang Bridge in Zhaoqing, China. The bridge is a continuous rigid frame bridge built in 2004 in the Guangdong province, China. It consists of seven spans with a total length of 808 m. The photograph and schematic diagram of its structure are presented in Figure 14. Properties of the bridge are summarized in Table 3. 

In order to demonstrate the sensitivity of DWPCA for damage detection of the full-scale structure, response data from different damage scenarios should be prepared. Since the bridge is in good conditions after the completion of construction stages, there are no damage events that could have generated unusual structural behavior. For the purposes of application of DWPCA on real structures, a full-scale FE model of the bridge is established. The strain responses under seasonal temperature variations presented in Figure 2 in Section 3 are obtained. Continuous structural health monitoring responses of four years at a sampling frequency of four measurements per day are collected.

**Figure 15:**
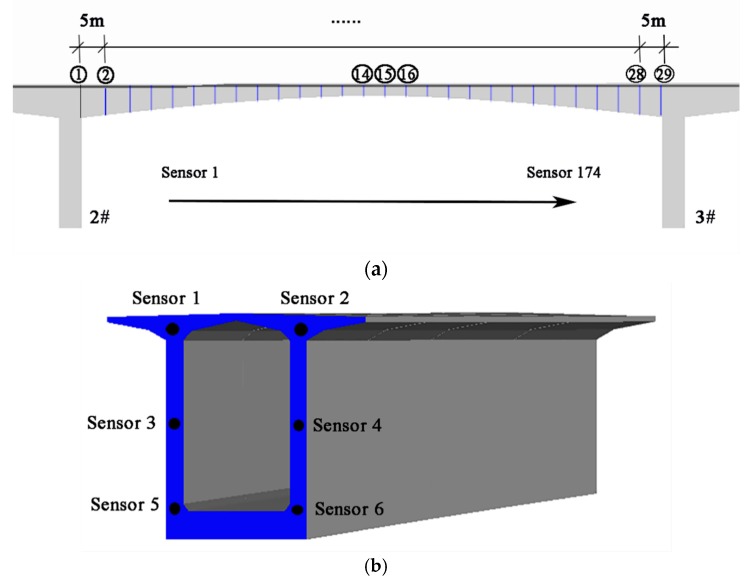
Spatial distribution of the sensors in the span between the 2# and 3# piers: (**a**) the arrangement of the monitoring section marked in blue along the bridge; (**b**) the arrangement of the sensors in the monitoring section.

**Figure 16:**
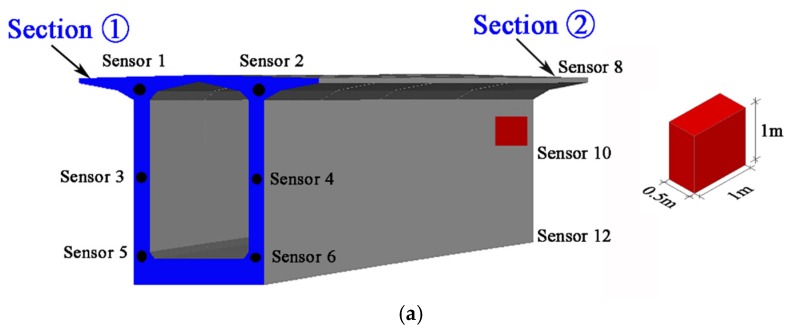

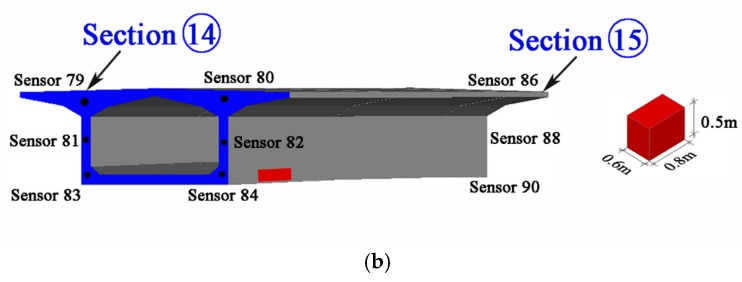
Damaged scenarios for evaluation of the damage detection algorithms: (**a**) damage with a stiffness reduction of 40% in the vicinity of Sensor 8 and Sensor 10; (**b**) damage with a stiffness reduction of 40% close to Sensor 84.

Local damage is assumed to be introduced in the span between the 2# and 3# piers of the bridge, as shown in Figure 14b. Sensors are embedded every 5 m along the bridge length, as shown in Figure 15a. The arrangement of the sensor locations on the top, webs and bottom of the girder box are given in Figure 15b. Note that there are 29 monitoring sections, and each section has six sensors installed in this span. Thus, there are 174 sensors in total and these are numbered from top to bottom, from left to right (Section ① to Section ㉙) in sequence. In the FE model, damage is assumed to be at a specific element of the bridge with a permanent stiffness reduction and is introduced at the beginning of the third year. Two different damage scenarios with different damage locations marked as red are shown in Figure 16a,b:

(1). Scenario A: Damage between Section ① and Section ② in the vicinity of Sensor 8 and Sensor 10, as shown in Figure 16a;
(2). Scenario B: Damage between Section ⑭ and Section ⑮ close to Sensor 84, as shown in Figure 16b.

Space windows which are related to the DSA should be determined. During the DSA analysis in this section, the parameter η in Equation (10) is equal to 5%. The $p_{0}$ in Equation (11) is set to be 60%. After simulations for a large number of damage scenarios, it was found that the DSA is more likely to lie within two neighboring monitoring sections that are located close to the damage. Especially, when the damage is located quite close to one monitoring section, the DSA is located in the vicinity of this section. The space windows considered herein contain sensors from two neighboring monitoring sections or from one section that is closest to the damage. Thus, for brevity of demonstration, only the following spatial windows are used for comparative studies between MPCA and the proposed DWPCA:

(a) Window A involving all the sensors: $\left[\begin{array}{cccc} 1 & 2 & \ldots & 174 \end{array}\right]$;
(b) Window B involving sensors from Section ① and Section ②: $\left[\begin{array}{cccc} 1 & 2 & \ldots & 12 \end{array}\right]$;
(c) Window C involving sensors from Section ⑭ and Section ⑮: $\left[\begin{array}{cccc} 79 & 80 & \ldots & 90 \end{array}\right]$;
(d) Window D involving sensors from Section ①: $\left[\begin{array}{cccc} 1 & 2 & \ldots & 6 \end{array}\right]$;
(e) Window E involving sensors from Section ②: $\left[\begin{array}{cccc} 7 & 8 & \ldots & 12 \end{array}\right]$;
(f) Window F involving sensors from Section ⑭: $\left[\begin{array}{cccc} 79 & 80 & \ldots & 84 \end{array}\right]$;
(g) Window G involving sensors from Section ⑮: $\left[\begin{array}{cccc} 85 & 86 & \ldots & 90 \end{array}\right]$.

Comparative studies of CEVs computed by MPCA ($\Delta \psi_{ij}^{M}$) and DWPCA ($\Delta \psi_{ij}^{DW}$), respectively, on damage detection for this bridge are presented as follows. Window A is still used in MPCA. Other windows including Window B to Window G will belong to the DWPCA method in the following demonstration.

**Figure 17:**
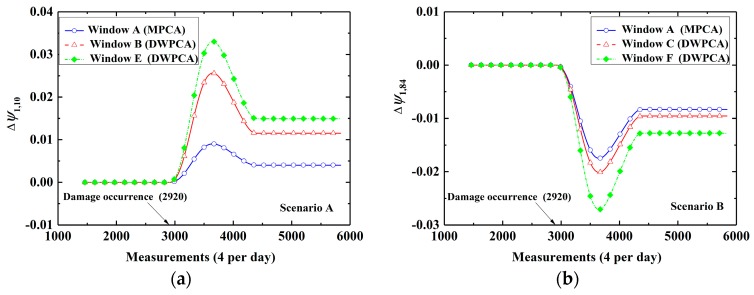
Evolution of the corresponding CEVs for different space windows: (**a**) Scenario A; (**b**) Scenario B.

Figure 17 shows the evolution of CEVs by MPCA and DWPCA upon application in two different damage scenarios. Similarly to that of the planar beam, there are no relative variations in the corresponding CEVs in the first two years since there is no damage. In addition, there exists a shift after damage occurrence in all scenarios when the time window involves responses from both damaged and healthy states. Then, the corresponding CEVs will become stable again after 4380 measurement events when responses within the time window are obtained from the damaged structure after 4380 measurements. Note that a more significant change of corresponding CEVs by the proposed DWPCA method with Window B, C, E, or F are observed in contrast to MPCA with Window A in both scenarios. Additionally, Windows E and F, which consist of sensors from only one monitoring section, perform better than Windows B and C, which contain sensors from two neighboring monitoring sections, when damage is located quite close to one monitoring section.

**Figure 18:**
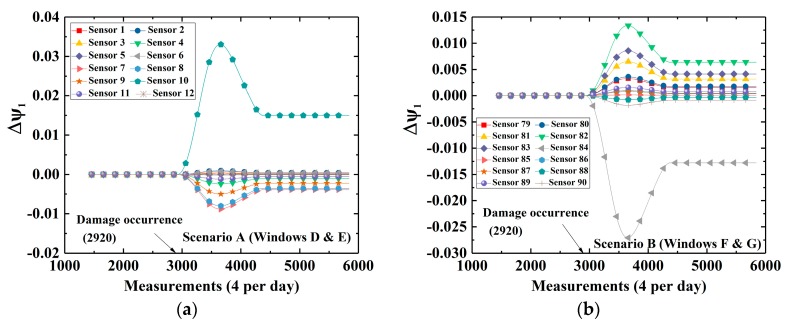
Time-variant CEVs with different space windows for Scenario A and B: (**a**) Windows D and E; (**b**) Windows F and G.

After the investigation of DWPCA in damage identification in the bridge, a closer look at all CEVs evolutions within a spatial window will be further explored for damage localization. Figure 18a shows the evolution of CEVs computed by DWPCA with Window D and E for Scenario A, in which the damage is located between Section ① and Section ②, close to Sensor 10. For Window D and E, as shown in Figure 18a, the CEVs corresponding to Sensor 7, Sensor 8, Sensor 9 and Sensor 10 shows an evident shift after damage occurrence as compared with other sensors which are located far from damage. This is due to the fact that Sensor 7, Sensor 8, Sensor 9 or Sensor 10 is the nearest to damage in the corresponding space window. For Scenario B in Figure 18b, as expected, the CEV related to Sensor 84 is the most evident because the damage is in the vicinity of Sensor 84. Figure 18b also indicates that the CEVs corresponding to Sensor 81, Sensor 82, and Sensor 83, which are close to damage, also have a remarkable shift after damage occurrence. It is seen that DWPCA can be used to localize damage with the aid of various space windows for complex engineering structures.

**Figure 19:**
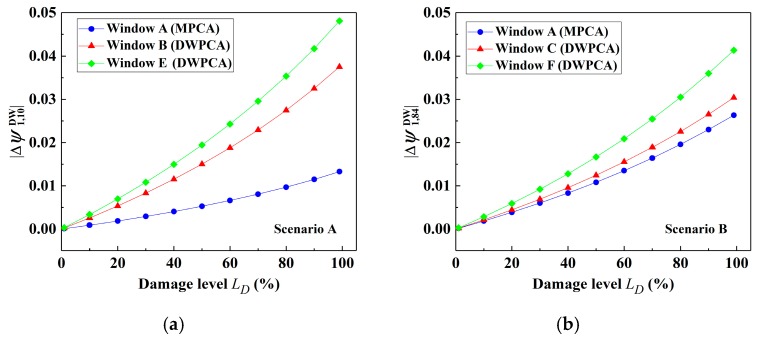
Variation of the absolute value of the CEVs with damage level by MPCA and DWPCA: (**a**) Scenario A; (**b**) Scenario B.

Based on the discussion in Section 4.3, the relationship between the damage level and stable absolute value of CEV after damage occurrence for a range of damage severities in simple beams can be quantitatively evaluated. Similarly, for Scenarios A, the damage level $L_{D}$ can be quantitatively evaluated in terms of $\left|\Delta \psi_{1.10}\right|$ by DWPCA with Window E in Figure 19a, as indicated by:

(15) $$L_{D}=1.25\ln\left(\left|\Delta \psi_{1.10}^{DW}\right|+0.037\right)+4.121$$

As for DWPCA with Window F for Scenario B, as presented in Figure 19b, the damage level $L_{D}$ can also be obtained from the calculated $\left|\Delta \psi_{1.84}\right|$ with the use of the following equation:

(16) $$L_{D}=1.25\ln\left(\left|\Delta \psi_{1.84}^{DW}\right|+0.032\right)+4.303$$

In summary, the proposed method DWPCA is demonstrated to be feasible for damage detection for large-scale structures. Results show that, similarly with the conclusion drawn in Section 4 for the planar beam, DWPCA has better performance in damage identification, damage localization and damage quantitative evaluation, as compared with the previous method MPCA. The is due to that the space windows used in DWPCA are capable of excluding damage-insensitive data from those sensors located far from the damage to enhance the damage detectability The proposed method is proven to have potential in applications for practical engineering.

## 6. Conclusions

This paper provides a novel effective method for structural damage detection by introduction of space and time windows in the traditional principal component analysis method. Due to the presence of the space window, the damage-insensitive data from those sensors located far from the damage are excluded in the analysis, and the damage detectability of the proposed method is improved in contrast to previous methods. Numerical results with a planar beam model demonstrate that the proposed method DWPCA, as compared with MPCA, improves the resolution for damage identification and is also quicker to detect damage after its occurrence. DWPCA is successful to detect minor damage with 0.1% stiffness reduction and identify damage 31 to 59 days earlier as compared with MPCA for a planar beam. With the aid of various space windows, the method is verified to have a better performance for damage localization as well. As for a quantitative evaluation of the damage severities from 0.1% to 99.9% for a planar beam, DWPCA proves to be more sensitive than previous methods. Finally, the proposed method is demonstrated to have good noise immunity and the result with a full-scale structure shows potential for applications in practical engineering. Further investigation will be focused on the feasibility of the proposed methodology to large-scale structures under more complicated loads such as real temperature variations and vehicle loads.

## Figures and Tables

**Table 1:** Detection resolution of moving principal component analysis (MPCA) and double-window PCA (DWPCA) for different scenarios.

Scenario	Detection Resolution in Damage Level (%)	
	MPCA	DWPCA
A	1	0.1
B	1	1
C	30	10
D	10	5

**Table 2:** Time to detect damage of MPCA and DWPCA for different scenarios.

Scenario	Stiffness Reduction (%)	Time to Detect Damage (Day)	
		MPCA	DWPCA
A	1	38.25	7.75
B	1	52.5	14
C	30	58.75	11.75
D	10	79	21

**Table 3:** Properties of each part of the Xijiang Bridge in Zhaoqing, China.

Parts		Material	Elastic Modulus (GPa)	Poisson Ratio
**Pier**	1#	Concrete	34.5	0.2
	2#–6#	Concrete	30.0	0.2
Bridge deck	Box girder	Concrete	32.5	0.2
	Non-pressed and pressed steel	Steel	195.0	0.3

## Abbreviations

The following abbreviations are used in this manuscript:

CEV	Component of the eigenvector variance
DSA	Damage-sensitive area
DWPCA	Double-window principal component analysis
FE	Finite element
MPCA	Moving principal component analysis
PCA	Principal component analysis
SHM	Structural health monitoring
